# Comparison of the Technological Application Potential of Functional Ingredients for the Meat Industry Based upon a Novel Fast Screening Tool

**DOI:** 10.3390/foods10092078

**Published:** 2021-09-02

**Authors:** Olivier Goemaere, Bart De Ketelaere, Jana Hanskens, Quinten Masijn, Cristina Pérez Santaescolastica, Ilse Fraeye

**Affiliations:** 1 Research Group for Technology and Quality of Animal Products, Department of Microbial and Molecular Systems, Leuven Food Science and Nutrition Research Centre (LFoRCe), KU Leuven Ghent Technology Campus, B-9000 Gent, Belgium; olivier.goemaere@kuleuven.be (O.G.); jana.hanskens@kuleuven.be (J.H.); quinten.masijn@kuleuven.be (Q.M.); cristina.perezsantaescolastica@kuleuven.be (C.P.S.); 2 KU Leuven, Faculty of Bioscience Engineering, BIOSYST—MeBioS, Kasteelpark Arenberg 30, B-3001 Leuven, Belgium; bart.deketelaere@kuleuven.be

**Keywords:** ingredient functionality, meat model systems, standardized methodology, cluster analysis

## Abstract

The application potential of functional ingredients for the meat industry is often assessed through different measuring tools, thereby making comparisons difficult. The aim of this study was to create valuable information about the performance of functional ingredients based upon standardized and comparable data gathered through a newly developed screening tool. Therefore, 25 ingredients, selected from different techno-functional classes, were characterized at 2 different dosages by means of the screening methodology. The tool itself consisted of a lean meat model and fatty liver-based system, representative of the finely minced and/or emulsified charcuterie market. A total of 23 different parameters were measured through both model systems, providing information concerning water and fat binding capacity, emulsification, and texture and structure formation. Through cluster analysis, the ingredients were assigned to groups, each with their own specific properties. The screening tool provided good descriptive and distinctive power concerning ingredient functionalities and offers the industry a clear overview of their application characteristics.

## 1. Introduction

Processed meat products often contain so-called techno-functional ingredients that are added to improve product quality, reduce formulation costs, satisfy consumer needs (texture, flavor, appearance, etc.), and to keep up with current market trends, such as clean label, fat reduction, and salt reduction. Balestra and Petracci [1] indicated that these ingredients can be divided into those that influence the functionality of the myofibrillar proteins (such as salt and phosphate) and those that form an extra system to increase water and fat binding and/or optimize product texture. This publication deals with the latter group, which can be further divided into several product classes, including starches, plant and animal proteins, dietary fibers (soluble and insoluble fibers, gums, …), and emulsifiers sensu stricto. In literature and through market survey, a lot of useful and scientific knowledge can be found with regard to their application in meat products. However, this information is subject to two drawbacks.

Firstly, ingredient manufactures often rely on water-based model systems to describe the characteristics of their products. Yet, it is well-known that ingredients often behave differently in a watery environment compared to a meat matrix. The importance of a meat matrix to measure ingredient functionality was described by Balestra and Petracci [1]. A food matrix is a much more complex medium, where multiple interactions between ingredients and other components (i.e., salts, lipids, and proteins) may occur, thus modifying the performance of the functional ingredient [2].

Many authors [1,3,4,5,6,7,8,9,10,11,12,13] researched and reviewed the potential of numerous techno-functional ingredients in different meat products. Despite good information available in these studies, this knowledge is subject to the second drawback. Standardized comparison between ingredients for specific quality characteristics such as water binding or texture development is difficult because these surveys were conducted on different meat matrices (differences in meat product class, composition of recipes, and processing conditions) and different analyzing techniques were applied. The development of a well-balanced, standardized, elementary meat matrix could therefore provide a good screening environment for the potential of functional ingredients for the meat industry, resulting in uniform and easily comparable scientific data.

Furthermore, a comprehensive literature study revealed that functional ingredients often contribute to similar quality characteristics (water and fat binding, structure formation, texture improvement) in meat products, despite their difference in nature, origin, or product class. Therefore, classification of ingredients (at specific dosages) based upon similar behavior rather than product class could be more beneficial or practical for the meat industry. As mentioned before, a standardized and uniform measuring tool is hereby of great value. Therefore, the goal of this study is to gather key information concerning the characteristics of 25 techno-functional ingredients, each applied at two dosages in a meat matrix based upon a newly developed screening tool. The obtained standardized and scientifically collected data are statistically processed to cluster ingredient x dosage combinations in groups exerting similar performances. This will lead to better insights and understanding of their functionalities, and thus, better estimation of the use of certain ingredient x dosage combinations in specific meat applications.

## 2. Materials and Methods

### 2.1. Experimental Setup

A lean meat model (LMM) and fatty liver-based model system (FLM) were developed and validated to serve as a standardized screening tool for the measurement of functional ingredient characteristics. They represent the wide range of both lean (cooked ham) and fatty heated meat products available in the market. Altogether, 23 different parameters (see following sections) were measured through the model systems.

In both LMM and FLM, a total of 25 ingredients were tested. The choice of ingredients was based upon a short inquiry of the Flemish meat industry and was mainly directed by their common practice. Ingredients were derived from different functional classes and fixed low (LD) and high dosage (HD) levels were defined for each class, based on ingredient product sheets and literature (Table 1). The LD of each ingredient class corresponds to the minimum concentration needed to improve product quality to some extent. The ingredient dosages were calculated relative to the total mass of meat raw materials (*Longissimus dorsi* muscle for LMM; liver and back fat for FLM) and water.

To match the scientific goals to the available experimental material and time, the experiments were optimized in such a way that replication was maximized for each treatment (i.e., ingredient x dosage combination), and this was the case for both model systems, taking into account the different ingredient classes that were considered (Table 1). In addition, blanks (4 replicates) of both LMM and FLM lacking any ingredient were included as a reference treatment. A total of 104 experimental runs, including the 4 blanks, were performed throughout the study, and this was applied to both the LMM and FLM model systems.

### 2.2. Manufacturing of Lean Meat and Fatty Liver-Based Model Systems

LMM and FLM model systems were prepared in the pilot plant of the research group ‘Technology and Quality of Animal Products’ (KU Leuven Ghent Technology Campus, Gent, Belgium). Raw materials (pork *L. dorsi* muscle, pork liver, and pork back fat) were purchased from a local supplier. The entire experiment was performed using a single homogeneous batch of each respective raw material. Back fat was cut into small cubes, and *L. dorsi* muscle and liver were ground through an 8 mm plate. All raw materials were each mixed thoroughly to obtain a large uniform batch of each raw material, packed as smaller portions into plastic bags and frozen (−18 °C) until use. The day before preparation of the specific model systems, raw materials were thawed overnight in a refrigerator at 4 °C. This approach allowed us to study only the impact of the ingredients without interference of potential variability in raw material characteristics.

LMM was composed of *L. dorsi* muscle (40 g/100 g) and ice-water (60 g/100 g), in which nitrite curing salt (1.6 g/100 g) and tetrasodium pyrophosphate (0.2 g/100 g) were dissolved in advance. Auxiliary ingredients were calculated relative to the total mass of meat raw materials (*L. dorsi* muscle) and water. The lean meat and brine were shortly mixed (10 s) at 2000 RPM before adding the ingredient under investigation (Table 1) to the Grindomix GM200 chopper (Retsch GmbH, Haan, Germany). The batter was further ground for approximately 5 min at 3500 RPM to a temperature of 12 °C.

After chopping, part of the meat batter was used directly to map dynamic viscoelastic properties (Section 2.3) and water binding parameters (Section 2.5). The residual meat batter was filled in 2 aluminum cans and heated for 90 min at 76 °C in a Rational ClimaPlus Combi oven (Rational Belgium SA, Zwijndrecht, Belgium), which corresponds to an industrial pasteurization process. Afterwards the cans were stored in a cool cell at 3 °C. Model systems were analyzed 5–6 days after processing with respect to cooking loss (Section 2.6), pH (Section 2.8), and textural properties (TPA, Section 2.9). Syneresis of the model systems was determined after 3 weeks of cold storage (Section 2.7).

The formulation of the FLM consisted of liver (15 g/100 g), pork back fat (50 g/100 g), and broth obtained by scalding the back fat (35 g/100 g). It was processed by first separately prechopping the raw liver for 8 min at 3500 RPM in a Stephan UM12 vertical cutter-mixer (Stephan Machinery GmbH, Hameln, Germany). The liver batter was stored at 4 °C until further processing. The back fat was scalded in boiling water during 20 min. Afterwards, the scalded fat together with the broth were added to the bowl cutter and chopped at 3500 RPM during 5 min. Temperature of the obtained fat emulsion reached 50–51 °C at the end of chopping. The liver batter, nitrite curing salt (1.6 g/100 g), and ingredient under investigation (Table 1) were added and mixed in the cutter during 3 min at 3500 RPM and 80% of vacuum. Nitrite curing salt was calculated relative to the total mass of meat raw materials (liver and back fat) and broth. Part of the batter was immediately analyzed to determine dynamic viscoelastic properties (Section 2.3) and emulsion stability (Section 2.4). The remainder of the batter was subsequently filled into cans, heated, cooled, and stored in a cool cell until further analyses. Thermal processing conditions and analyses on finalized product (with the exception of syneresis) were the same as described for LMM.

Both recipes of the described model systems have a quite critical composition, meaning that they contain substantially more water and/or fat than regular meat products. These recipes were developed to induce poor product quality, allowing us to measure the effect of the different ingredients and compare them more easily.

### 2.3. Dynamic Viscoelastic Properties

Rheological measurements of both raw LMM and FLM batters were executed using an AR2000ex stress-controlled rheometer (TA Instruments, New Castle, DE, USA) equipped with a 40-mm parallel plate. The upper and lower plate were crosshatched to prevent slippage of the samples. The gap between the plates was set at 1000 μm. The AR2000ex was equipped with a Peltier temperature control system and upper heated plate (TA Instruments) to control the sample temperatures precisely.

Stress sweeps were executed at a temperature of 14 °C and 40 °C for LMM and FLM system, respectively. Oscillation stress was increased between 0.1 and 1000 Pa at constant frequency of 1 Hz to determine the linear viscoelastic region (LVR). The ‘storage’ and ‘loss’ modulus (*G′*, measurement of elastic characteristics and *G″*, measurement of viscous characteristics respectively) were directly obtained from the Rheology Advantage Data Analysis, v. 5.7.0 software (TA Instruments, New Castle, DE, USA). The LVR represents the stress range within which both *G′* and *G″* are independent of the imposed stress amplitude and is determined according to Glorieux et al. [14]. The length of the LVR and the constant value of G′ within the LVR of both LMM (*LVR-LMM, G′-LMM*) and FLM (*LVR-FLM, G′-FLM*) are expressed logarithmically and used in the cluster analysis (Section 2.10).

**Figure 1:**
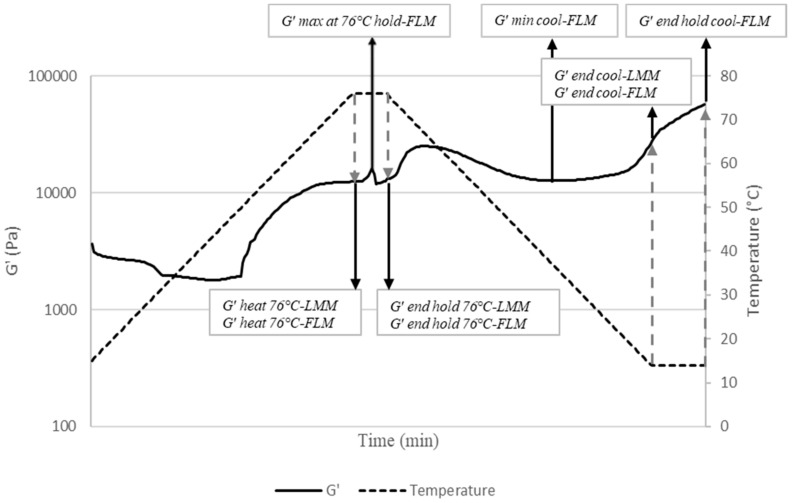
Rheological measurement, composed of a concatenation of temperature ramps and time sweeps, representing thermal process simulation of both LMM and FLM. Simulation starting temperatures are 14 °C and 40 °C for LMM and FLM, respectively. Second time sweep step at 14 °C is only executed for FLM. Values of G′ derived from graph and used as parameters in cluster analysis include: *G′ heat 76 °C-LMM* and *G′ heat 76 °C-FLM* referring to G′ at end of first temperature ramp (76 °C) for both model systems; *G′max at 76 °C hold-FLM* referring to maximum measured value of G′ during first time sweep (76 °C) of FLM; *G′ end hold 76 °C-LMM* and *G′ end hold 76°C-FLM* referring to G′ at end of first time sweep (76 °C) for both model systems; *G′ min cool-FLM* referring to minimum value of G′ during second temperature ramp of FLM; *G′ end cool-LMM* and *G′ end cool-FLM* referring to G′ at end of second temperature ramp (14 °C) for both model systems; *G′ end hold cool-FLM* referring to G′ at end of second time sweep (14 °C) of FLM.

Temperature ramps and time sweeps were executed to examine structural changes of both LMM and FLM batters during heating and sequential cooling. The applied temperature profile represents the manufacturing process of industrially processed lean and fatty (emulsified) meat products. The following profile was applied: (1) heating from 14 °C and 40 °C for LMM and FLM, respectively, to 76 °C at a constant heating rate of 2 °C/min; (2) isothermal time sweep at 76 °C for 3 min; (3) cooling from 76 °C to 14 °C at a constant cooling rate of 2 °C/min; (4) isothermal time sweep at 14 °C for 10 min (only for FLM). The entire process is illustrated in Figure 1, in which G′-values of interest regarding possible influence of added functional ingredients in both model systems are explained. These parameters were expressed logarithmically and used in the cluster analysis (Section 2.10). Oscillation measurements during the entire process were performed at a fixed frequency of 1 Hz and a constant strain of 0.010 for FLM and 0.025 for the LMM (within the LVR). Rheological measurements were executed in duplo for each experimental run regarding LMM batters, while for FLM, they were performed in singular due to instability of the freshly produced batter.

### 2.4. Emulsion Stability

Emulsion stability of FLM was determined immediately after batter preparation according to Glorieux, Goemaere, Steen and Fraeye [14] with slight modifications. Summarized, emulsion stability is expressed as drip loss upon heating (30 min, 75 °C) and centrifugation at 4234× *g* by means of a Universal 320 R centrifuge (Andreas Hettich GmbH & Co. KG, Tuttlingen, Germany) at 25 °C for 3 min of a preweighed amount of raw batter. The percentage of total expressible fluid (*TEF-FLM*) was expressed as follows:

(1) $$\mathrm{TEF}-\mathrm{FLM}\;(\%)=\frac{drip\;loss}{initial\;weight\;sample}\times 100$$

In addition, the relative amount of water next to the fat in the drip was determined. Therefore, drip loss after centrifugation was collected and weighed before and after drying in a Typ U 40 oven (Memmert, Schwabach, Germany) for 24 h. The relative amount of water in the drip loss was expressed as follows:

(2) $$\mathrm{Relative}\;\mathrm{amount}\;\mathrm{of}\;H_{2}O\;\mathrm{in}\;\mathrm{drip}-\mathrm{FLM}\;(\%)\;=\;\frac{drip\;loss\;before\;drying-drip\;loss\;after\;drying}{drip\;loss\;before\;drying}\times 100$$

*TEF-FLM* and *Relative amount of H_2_O in drip-FLM* were determined *in triplicate* for each experimental run and used as parameters in the cluster analysis.

### 2.5. Drip Loss

The drip loss of the raw batter of LMM was measured immediately after batter preparation (*Drip loss cold-LMM*). The determination was executed in duplo for each experimental run by centrifuging 30 *g* of meat batter at 9526× *g* (9000 rpm) for 5 min at 25 °C, after which the separated fluid was weighed. *Drip loss cold-LMM* was expressed as follows:

(3) $$\mathrm{Drip}\;\mathrm{loss}\;\mathrm{cold}-\mathrm{LMM}\;(\%)=\frac{drip\;loss}{initial\;weight\;sample}\times 100$$

Determination of the drip loss of the batter of LMM upon heating (*Drip loss hot-LMM*) is similar to the cold procedure, albeit that before centrifugation at 470× *g* (2000 rpm) for 1 min at 25 °C the samples were heated at 75 °C for 30 min in a KK 2800 smoke-air cooker (Kerres Anlagen systeme GmbH, Backnang, Germany). *Drip loss hot-LMM* was determined *in triplicate* for each experimental run and expressed as follows:

(4) $$\mathrm{Drip}\;\mathrm{loss}\;\mathrm{hot}-\mathrm{LMM}\;(\%)=\frac{drip\;loss}{initial\;weight\;sample}\times 100$$

*Drip loss cold-LMM* and *Drip loss hot-LMM* were used as parameters in the cluster analysis.

### 2.6. Cooking Loss

Cooking loss of LMM (*CL-LMM*) was determined in duplo for each experimental run according to Perez-Santaescolastica et al. [15]. Cooking loss was calculated as follows and applied as a parameter in the cluster analysis:

(5) $$\mathrm{CL}-\mathrm{LMM}\;(\%)=\frac{drip\;loss}{initial\;weight\;sample}\times 100$$

### 2.7. Syneresis

LMM was sliced and MAP packed (70% N_2_ and 30% CO_2_) in plastic trays. The trays were stored upright under an angle of 45° in a cool cell at 3 °C. After 3 weeks of storage, the drip loss was collected and used as a measurement for the syneresis of the sample. *Syneresis-LMM* was determined in duplo for each experimental run and expressed as follows:

(6) $$\mathrm{Syneresis}-\mathrm{LMM}\;(\%)=\frac{drip\;loss}{initial\;weight\;sample}\times 100$$

### 2.8. pH

pH of the model systems was measured as described in Glorieux, Goemaere, Steen and Fraeye [14]. A total of 4 and 6 pH-values were obtained for each experimental run regarding LMM and FLM, respectively. *pH-LMM* and *pH-FLM* were used as parameters in the cluster analysis.

### 2.9. Texture

Hardness of the model systems was determined using a Lloyd Texture Analyzer Model LF plus (Lloyd Instruments Ltd, Bognor Regis, UK) and expressed as the maximum force (N) to penetrate the sample, as described in Perez-Santaescolastica, Goemaere, Hanskens, Lorenzo and Fraeye [15]. A total of 4 and 9 hardness values were obtained for each experimental run regarding LMM and FLM, respectively. *Hardness-LMM* and *Hardness-FLM* were used as parameters in the cluster analysis.

### 2.10. Cluster Analysis

To cluster all ingredients with the given concentration (high/low), a hierarchical cluster analysis was performed based on the 23 above-described quality parameters. Mean values of each parameter per ingredient x dosage combination were expressed relative to their corresponding average blank value by dividing both and used for further statistical processing. To define the clusters, Ward’s minimum variance method was used [16]. Decision on the number of clusters was based on the Cubic Clustering Criterion (CCC) that is proposed in the JMP Pro 14 software.

All statistical analyses were performed in JMP Pro 14 (SAS Institute, Cary, NC, USA).

## 3. Results

### 3.1. Overview Hierarchical Clustering

**Figure 2:**
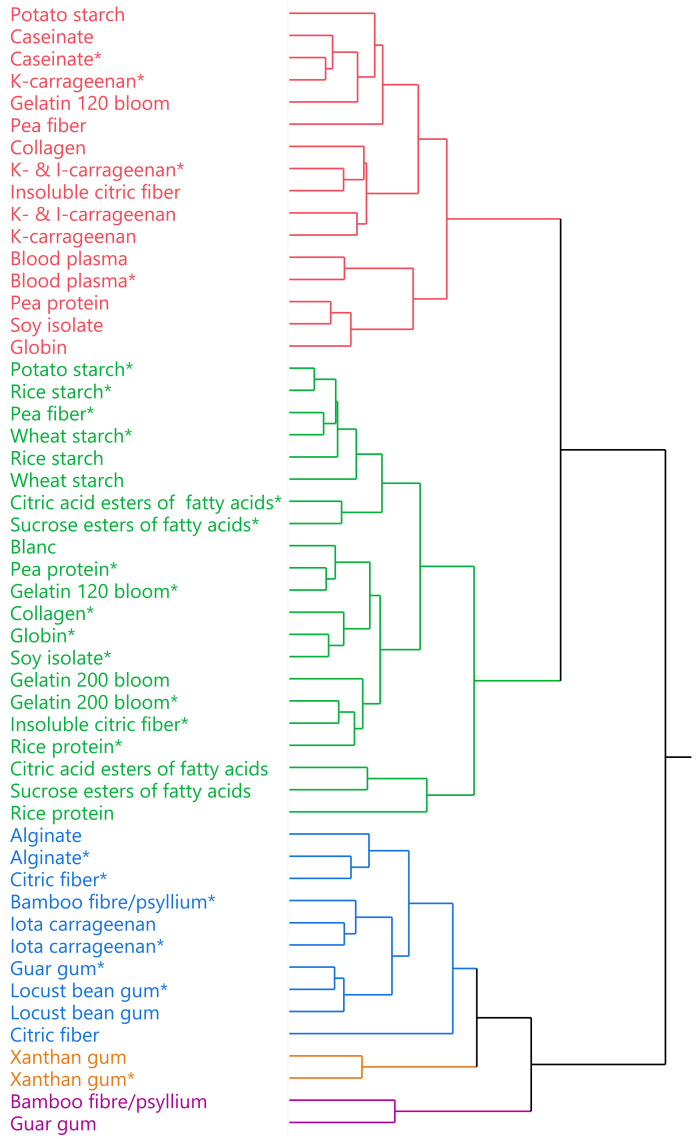
Dendrogram based on hierarchical cluster analysis of 25 screened ingredients at 2 dosages. Ingredient x dosage combinations occurring in same color have similar properties and belong to same cluster. ‘*’ means ingredient is tested at low dosage. Dosage is product class-dependent, as indicated in Table 1.

Figure 2 reveals the hierarchical clustering of the tested ingredient x dosage combinations. To construct the clustering, all 23 measured parameters on LMM and FLM were considered. Five main clusters, indicated by different colors, can be distinguished based on the CCC. Each cluster groups ingredient x dosage combinations showing similar and unique behavior. In the paragraphs below, each cluster is described, and explanations are formulated to comprehend the differences between them. The green cluster containing the blank sample is discussed first and set as a reference to evaluate the others.

### 3.2. Green Reference Cluster

The green cluster includes the blank (reference) to which no ingredients were added. Hence, ingredient x dosage combinations in the green cluster represent samples which have limited impact on the parameters measured compared to the blank sample. Of the five clusters identified, the green cluster groups the highest amount of ingredients, mainly ingredients at LD. The LD of each ingredient class is established, as mentioned before, based upon literature and available product sheets and corresponds approximately to the minimum dosage needed to improve product quality to some extent. As a consequence, the impact of LD ingredients on parameters measured is rather small compared to the blank.

Closest to the blank sample are mainly collagen-derived proteins and plant proteins at LD. The contribution of gelatins to water binding in heated meat products is rather limited mainly due to their lack of gelling capacity at high temperature. During heating, gelatin solubilizes in the watery phase [17] and can migrate out of the product together with the cooking loss. Since the gelatin network is thermo-reversible [18,19], it can partially reappear during cooling outside the product matrix as gel deposits. Its contribution to water binding and texture/structure of standard heated meat products is therefore rather limited.

In general, plant proteins have less pronounced functional properties compared to that of animal proteins in meat products. Moreover, an effective replacement of animal proteins requires certain technological innovations. New extraction and drying technologies are proposed to ameliorate functional characteristics of plant proteins such as pea protein [1,18]. Adding them at LD apparently often resulted in a limited effect on the parameters measured.

Somewhat further away from the blank sample are starches and emulsifiers. The appearance of pea fiber in that group can be easily explained due to the relatively high amount of starch in the composition of the ingredient, according to the manufacturer (not shown). All starches screened in the experiment are (functional) native starches. In general, the functionality of native starches is rather restricted compared to chemically modified starches, which could explain their occurrence in the green cluster. Nowadays more and more starch producers have found innovative solutions to create ‘native functional’ starches by using physical processes with comparable properties to chemically modified ones [1,9]. The presumable contribution of emulsifiers sensu stricto to product characteristics, most likely fat stabilization, is only possible in fat-containing products. In the LMM, they do not exert any function at all. This may partly explain the presence of emulsifiers in the green cluster. Emulsifiers and rice protein at HD are at the edge of the green cluster away from the blank. This can be explained by their negative impact on measured product parameters. Rheological thermal process simulations revealed interference of these ingredients with the formation of the liver gel network during heating (low *G′ heat 76 °C-FLM*). As a consequence, an inferior stabilization of fat, expressed as a high increase of *TEF-FLM*, occurred. In case of emulsifiers, these phenomena can be explained through the different theories of fat stabilization in meat batters. The first theory states that stabilization of fat results from the formation of an interfacial protein film (IPF) around the fat globule, while the second one claims that fat globules are physically entrapped within the protein matrix [13]. Meat emulsions, like the FLM, are characterized by solubilized liver proteins encapsulating the fat within the matrix [20]. During heating, the emulsion is stabilized by the formation of protein cross-links between proteins in the IPF and the matrix proteins. In contrast, when nonprotein emulsifiers such as mono- and diglycerides are added, these emulsifiers can adsorb at the surface of the fat globules in preference to the liver proteins and cause a reduction in protein-lipid interactions. In this case, crosslinking of the IPF to the protein matrix is usually absent, and as a consequence, the continuous protein network may be altered, leading to an increase in fat loss [13]. The negative impact of rice protein on formation of the liver protein network and subsequent increase in drip loss can possibly be attributed to its damaging and weakening effect on the gel-forming ability of the liver proteins. Gu et al. [21] observed similar behavior when rice proteins were added to silver carp surimi gels. They presumed this was caused by impact of the rice proteins on the cross-linking abilities between myofibrillar proteins, consequently disrupting the fish gel matrix. Lin et al. [22] also noticed disruption of the microstructure of a myofibrillar protein gel upon addition of a mixture of rice and peanut protein isolate.

### 3.3. Red Cluster

**Figure 3:**
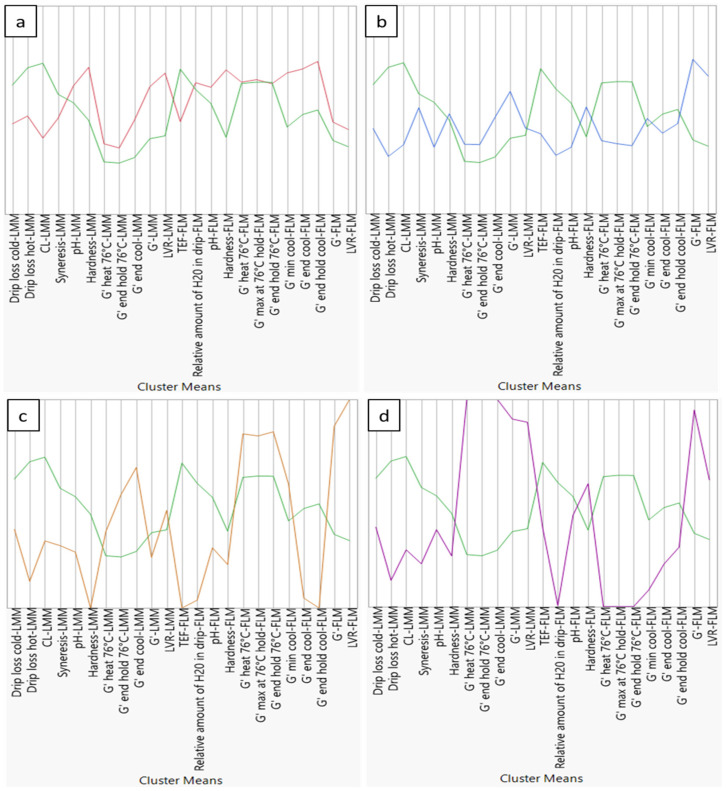
Parallel coordinates plots to provide an overview of 5 cluster means consisting out of 23 quality characteristics determined through developed lean meat model and fatty liver-based model. (**a**) Represents comparison of the ‘green reference cluster’ means vs. red cluster means; (**b**) represents comparison of ‘green reference cluster’ means vs. blue cluster means; (**c**) represents comparison of ‘green reference cluster’ means vs. orange cluster means, and (**d**) represents comparison of ‘green reference cluster’ means vs. purple cluster means. Explanation on used abbreviations can be found in Section 2 (Materials and Methods) and Figure 1. Axes range from two standard deviations above and below mean, where standard deviation and mean are computed for raw data.

Many ingredients in the red cluster are only included at HD, while their LD counterparts are located in the green ‘reference’ cluster, meaning that for these ingredients higher dosages usually have greater impact on the parameters measured. Figure 3a shows for each parameter the mean value over all ingredient x dosage combinations included in the red cluster compared to that of the green ‘reference’ cluster. In general, there is an improvement of all measured parameters for both model systems. Water-binding parameters of LMM show a decrease in cooking loss and drip loss of the raw and heated meat batter, and a small reduction of syneresis. Furthermore, hardness and rheological parameters of LMM batter before and during heating and subsequent cooling illustrate higher values indicating a firmer product structure. Similar conclusions, such as smaller drip losses and increased product structure, can be drawn from FLM. This cluster is further defined by ingredients which generally increase product pH to a small extent. Hereby, pH shifts away from the iso-electric point of the myofibrillar proteins, creating a greater net charge and repulsion between proteins [14]. It could also contribute to the improved water binding capacities of both model systems, but this is doubtful due to the rather small increase of pH.

The red cluster is populated with several proteins and more specifically animal proteins with good warm gelling and emulsifying properties such as globin, blood plasma, and caseinate [2,3,23,24,25,26,27]. The latter two already alter product quality at LD. Plant proteins such as soy isolate and pea protein can establish similar behavior, but only at HD. Another group of ingredients observed in the red cluster are (combinations of) κ-carrageenans, both at LD and HD. κ-carrageenan is widely used in the meat industry for its gelling properties and corresponding water-binding and structure-improving capabilities [28,29,30,31]. Several authors [28,32] stated that increased gel strength is probably not caused by molecular interaction between carrageenan and proteins. Verbeken et al. [33] claim this is probably due to the presence of carrageenans in the interstitial spaces of the protein network, where they form gel fragments upon cooling and therefore retain water. Impact of κ-carrageenan on hardness is higher compared to that of ι-carrageenan [34]. This can possibly be related to the fact that κ-carrageenan can form brittle gels, while ι-carrageenan forms elastic gels [28,31]. The different behavior between carrageenan types may also explain the difference in cluster classification for both ingredients. The behavior of (combinations of) κ-carrageenans is similar to the above-described protein performance and exactly what defines the red cluster. These findings contribute to the justification of the developed meat model systems as a screening instrument for functional ingredients and the choice of cluster analysis as statistical tool for data processing.

Potato starch and pea fiber at HD also show similar behavior compared to proteins and carrageenans, most likely because starch gelatinization during heating results in inclusion of water and increase of product structure. Potato starch has some advantages compared to other native starches, such as low gelatinization temperature (60–65 °C), high water binding capacity, and high viscosity. The combination of these attributes makes it an attractive ingredient for meat processing [3].

### 3.4. Blue Cluster

The blue cluster mainly contains ingredients such as gums and fibers with a soluble component. Most of those ingredients are present in the cluster both at LD and HD, indicating that small dosages already influence product quality. On the other hand, ingredients at HD have a more pronounced effect on certain parameters (individual results not shown) but this does not result into their classification in another cluster, suggesting that the impact of increased dosage on product parameters is rather limited. Figure 3b shows the comparison between cluster means of the green ‘reference’ and blue cluster. The unique character of this cluster is first determined by a strong water binding capacity. This was noticed in both LMM and FLM. Additionally, a shift in composition of the drip loss of FLM can be observed. The relative amount of water in the drip is substantially lower compared to that of the green ‘reference’ or red cluster. This is probably related to the rather strong water binding capacity and increase of batter viscosity caused by the ‘blue cluster ingredients’. The impact on structure of the raw and heated batter, especially of FLM, differs substantially from the green and red cluster. Structure (*G′-FLM*) and structure strength (*LVR-FLM*) of the raw batter are increased, while on the other hand, a rather strong negative impact on the formation of the liver network during heating is observed (*G′ max at 76 °C hold-FLM*).

Gums and fibers with a large proportion of soluble components are characterized by a thickening or weak gelling character, and therefore greatly improve water-binding capacity. These properties result from an equilibrium between water–gum interactions and intermolecular forces (i.e., hydrogen, hydrophobic and electrostatic bonds), which leads to the formation of aggregates or 3D matrix structures [1,12]. According to Tarté [4], fibers containing a higher level of soluble fibers, as was the case with the examined citric fiber and mixture of bamboo fiber and psyllium (due to the latter) [35,36], can boost water absorption, but tend not to have the oil/fat-binding capability of the higher insoluble fraction varieties. This could possibly explain the shift in composition of the drip loss for FLM.

The impact on structural parameters and protein network formation by ingredients in the blue cluster is described by several other authors. Whiting [37] stated that addition of guar could possibly disrupt the protein–protein gel network, which negatively affected gel strength as seen for FLM. Ramírez et al. [38] indicated a decrease in shear stress when locust bean gum is added to surimi gels. On the other hand Montero et al. [39] noticed an increase in breaking deformation and work of penetration when locust bean gum is added to fish proteins gels. They attributed this to the water-holding of the gum resulting in a more deformable gel. The same authors observed changes in myofibrillar gel flexibility and its breaking deformation by use of ι-carrageenan. In the current research, the addition of ι-carrageenan interfered with protein network formation, as seen by thermal processing simulation in FLM (*G′ max at 76 °C hold-FLM*). Several authors investigated the impact of alginate on structure and texture of meat products and protein gels. Sarteshnizi, Hosseini, Mousavi Khaneghah and Narges [28] suggested that alginate could change the physical state of proteins and affect texture of meat products. Furthermore Xiong and Blanchard [40] found a reduction in the gelling capacity of suspensions containing several combinations of salt-soluble proteins and alginate. They claim this could possibly be explained through interference in the protein network at gelling point. It is also probable that alginate has some influence on the electrostatic bonding, leading to a lower gel firmness in the final gelled product. The latter was especially noticeable in FLM as *G′ end cool-FLM* was much lower compared to that of the blank.

### 3.5. Orange Cluster

This cluster is solely occupied by xanthan at both dosages. As seen in Figure 3c, the orange cluster represents a very high water-binding capacity for both model systems, an atypical impact on structure formation observed during thermal process simulation, and a detrimental impact on product hardness compared to that of the green ‘reference’ cluster. The unique property of xanthan is the ability to increase viscosity of a liquid already at very low concentrations [30,41]. Sánchez, Bartholomai and Pilosof [12] concluded xanthan is very good at holding water within the meat matrix and stabilizing emulsions. Montero, Hurtado and Pérez-Mateos [39] noticed a great impact of xanthan addition on the structure of fish gels; it led to a decrease in gel forming capacity of the myofibrillar proteins as seen in the current research (*G′ max at 76 °C hold-LMM* and *FLM*). According to the authors, this was related to the presence of xanthan in the interstitial spaces of the matrix, where its high molecular weight probably hindered formation of the protein matrix. Also, its construction of large cavities in the network could be linked to the impact on structure. On the other hand Ramírez, Barrera, Morales and Vázquez [38] attributed the negative effects of xanthan to its anionic nature. Myofibrillar proteins are negatively charged at pH values above their isoelectric point, which is generally the case in meat and fish products, causing a repulsive effect with xanthan. This antagonistic effect weakens the protein network.

### 3.6. Purple Cluster

The purple cluster contains only two ingredient x dosage combinations: guar and the mixture bamboo and psyllium both at HD. The main reason only few ingredients are present in this cluster is, like in the orange cluster, the rather extreme behavior of the samples as seen in Figure 3d. Especially the evolution of G′ during thermal processing differed substantially compared to other ingredient x dosage combinations, as observed through extreme high and low values of *G′ end hold 76 °C*- for LMM and FLM, respectively. Despite the impact on product structure formation during processing, hardness of both LMM and FLM is not greatly affected, in contrast to xanthan (orange cluster). Although both orange and purple clusters exhibit ingredient x dosage combinations with divergent behavior, the developed model systems can still distinguish them. The intrinsic characteristics of psyllium, such as its extremely high water-binding and gel properties could explain the position of this ingredient in the purple cluster [4].

## 4. Conclusions

The developed toolbox consisting of a lean meat and fatty liver-based model system was a trustworthy measuring instrument to obtain standardized and comparable data concerning intrinsic ingredient functionalities. Hierarchical cluster analysis could create an efficient overview of ‘ingredient x dosage combinations’ based upon similar behavior, rather than product class, while maintaining a great distinctive power. It revealed 5 main ingredient clusters, each with their own unique characteristics. This new approach will help the meat industry and its partners in making the right choice of ingredient for specific meat applications based on scientific, standardized, and comparable data. Ingredient manufacturers and suppliers can also call on this screening tool to reveal the intrinsic characteristics of newly developed ingredients and compare them with what is available in the market. In future work, the obtained knowledge on model system level will be translated to several types of industrial meat products. The parameters measured in the model systems will be linked to quality characteristics of meat products on industrial scale.

## Figures and Tables

**Table 1:** Overview of screened ingredients including product names, classes, and tested dosages within each product class (low and high dosage, LD and HD, respectively). Abbreviations used: κ (Kappa), ι (Iota).

Product Class/Tested Dosage (LD–HD)	Product Name
Animal protein 1–2%	Collagen
	Gelatin 120 bloom
	Gelatin 200 bloom
	Blood plasma
	Globin
	Caseinate
Vegetable protein 1–2%	Pea protein
	Rice protein
	Soy isolate
Starch 1.5–3%	Potato starch
	Rice starch
	Wheat starch
(in)Soluble fiber 1–3%	Insoluble citric fiber
	Citric fiber
	Bamboo fiber/psyllium
	Pea fiber
Gum 0.5–1%	Guar gum
	Xanthan gum
	Locust bean gum
	Alginate
	κ-carrageenan
	κ-ι-carrageenan
	ι-carrageenan
Emulsifier sensu stricto 0.3–1%	Citric acid esters of fatty acids
	Sucrose esters of fatty acids
